# Individual Substitution Mutations in the AID C Terminus That Ablate IgH Class Switch Recombination

**DOI:** 10.1371/journal.pone.0134397

**Published:** 2015-08-12

**Authors:** Tatenda Kadungure, Anna J. Ucher, Erin K. Linehan, Carol E. Schrader, Janet Stavnezer

**Affiliations:** Department of Microbiology and Physiological Systems, Univ. of Massachusetts Medical School, Worcester, MA, 01605, United States of America; Institut de Recherches Cliniques de Montréal (IRCM), CANADA

## Abstract

Activation-induced cytidine deaminase (AID) is essential for class switch recombination (CSR) and somatic hypermutation (SHM) of Ig genes. The C terminus of AID is required for CSR but not for SHM, but the reason for this is not entirely clear. By retroviral transduction of mutant AID proteins into *aid* ^*-/-*^ mouse splenic B cells, we show that 4 amino acids within the C terminus of mouse AID, when individually mutated to specific amino acids (R190K, A192K, L196S, F198S), reduce CSR about as much or more than deletion of the entire C terminal 10 amino acids. Similar to ΔAID, the substitutions reduce binding of UNG to Ig Sμ regions and some reduce binding of Msh2, both of which are important for introducing S region DNA breaks. Junctions between the IgH donor switch (S)μ and acceptor Sα regions from cells expressing ΔAID or the L196S mutant show increased microhomology compared to junctions in cells expressing wild-type AID, consistent with problems during CSR and the use of alternative end-joining, rather than non-homologous end-joining (NHEJ). Unlike deletion of the AID C terminus, 3 of the substitution mutants reduce DNA double-strand breaks (DSBs) detected within the Sμ region in splenic B cells undergoing CSR. Cells expressing these 3 substitution mutants also have greatly reduced mutations within unrearranged Sμ regions, and they decrease with time after activation. These results might be explained by increased error-free repair, but as the C terminus has been shown to be important for recruitment of NHEJ proteins, this appears unlikely. We hypothesize that Sμ DNA breaks in cells expressing these C terminus substitution mutants are poorly repaired, resulting in destruction of Sμ segments that are deaminated by these mutants. This could explain why these mutants cannot undergo CSR.

## Introduction

After activation by immunization or infection, B cells undergo both Ig class switch recombination (CSR) and somatic hypermutation (SHM), which together result in the production of antibodies with improved ability to remove the immunogen or pathogen that induced the response. CSR exchanges the μ heavy chain constant (C_H_) regions for δ, γ, ε, or α C_H_ regions, altering the effector functions of the antibody without changing its antigen specificity. SHM is a process that introduces mutations into variable [V(D)J] regions of heavy and light chains, and combined with B cell selection, results in increased affinity for the antigen. CSR and SHM are both instigated by activation induced cytidine deaminase (AID), which deaminates cytosines (dC) converting them to uracils (dU) in the Ig heavy chain switch (S) regions and in the recombined V(D)J gene segments, respectively [1,2]. In order to lead to CSR, which generally occurs by non-homologous end-joining (NHEJ), the dU’s are converted to DSBs by the actions of both the base excision repair (BER) and mismatch repair (MMR) pathways [3,4]. Specifically, uracil DNA glycosylase (UNG) excises the dU base, leaving an abasic site, and AP endonucleases (APE1/2) nick the abasic site to create a single-strand DNA break (SSB) [2,4,5]. If the SSBs on opposite strands are sufficiently near they form DSBs. Alternatively, the MMR proteins, Msh2-Msh6, recognize the U:G mismatch, and recruit exonuclease which can resect from a SSB on one strand to a SSB on the other strand, thus creating a DSB [3,6,7]. Although UNG and APE2 also participate in SHM [2,8], DSBs are not required for SHM. AID-induced mutations at C:G bp are mostly generated by replication across the dU, or across the abasic site produced by UNG. Mutations at A:T bp are mostly dependent upon Msh2-Msh6 recognizing the U:G mismatch, which leads to error-prone repair initiating at SSBs [8–12].

Although still not completely understood, it has been known for several years that the C terminal 8–17 amino acids of AID are required for CSR but not for SHM [13–15]. This is not due to the importance of the C terminus for targeting AID to S regions, as cells expressing AID that lacks the last 10 amino acids (ΔAID) have been reported to have normal levels of Sμ region mutations [15], and also normal levels of S region DSBs [16–18]. These results suggest that the AID C terminus is important for the repair/recombination step in CSR, consistent with demonstrations that the C terminus is required for recruitment of NHEJ proteins to S regions in cells undergoing CSR [19,20]. Also, the C terminus has a Crm1-dependent nuclear export signal, hence ΔAID accumulates in nuclei where AID is rapidly degraded [21,22]. However, poor nuclear export does not explain the CSR deficiency of ΔAID [23,24]. It also does not prevent ΔAID from functioning in SHM.

As shown by chromatin immunoprecipitation (ChIP), the C terminus is important for recruiting (or for increasing the binding affinity of) both UNG and Msh2-Msh6 to S regions [17–19,25]. This implies that DSB formation might be less efficient in cells expressing ΔAID. Furthermore, deletion of the *msh2* or *msh6* genes has no effect on CSR in cells expressing ΔAID, unlike the reduced CSR observed in cells expressing full-length (FL)-AID [17]. Thus, the finding that DSBs are as frequent in ΔAID-expressing cells as in cells expressing FL-AID suggests that S-S recombination might be delayed in these cells, allowing DSBs to accumulate. This is supported by results showing that in the presence of DNA, AID interacts with the NHEJ protein DNA-PKcs, dependent on the AID C terminus [26]. AID has also been shown to be involved in recruiting several NHEJ proteins: Ku70, Ku80, XRCC4, 53BP1, ATM, Nbs1, γH2AX, and DNA-PKcs to S regions, also dependent upon the C terminus [19,20]. Furthermore, in cells expressing ΔAID, S-S junctions show increased lengths of junctional microhomology, which is typical of cells lacking NHEJ proteins [18–20,27]. The inability of ΔAID to recruit UNG and MMR to S regions could result in sparse SSBs, leading to DSBs that have long ss tails and therefore are inappropriate for NHEJ, resulting in aberrant S-S junctions [18–20,27], and reducing the recruitment of NHEJ proteins. The lack of participation of NHEJ proteins in CSR does not entirely explain the CSR deficiency in cells expressing ΔAID, because CSR can occur quite efficiently in the absence of NHEJ proteins, using microhomology-mediated/alternative EJ [28,29].

How AID might help to recruit MMR, UNG, and NHEJ proteins to S regions, dependent upon its C terminus, is unknown. There is no evidence that the interactions with AID are direct. Attempts to detect direct binding between AID and Msh2 or UNG [17] or DNA-PKcs failed [26]. Other investigators have attempted to identify proteins in cell extracts that interact with full-length AID in the absence of DNA, and numerous proteins have been identified, including Msh2, Msh6, DNA-PKcs, and APE1, but which proteins might be involved in recruiting them to AID are unknown [30,31]. Previous studies have identified individual amino acids within the C terminus that are important for CSR [16,19,21,23,24,32], and here we extend these studies by analyzing the effects of specific mutations of 4 amino acids in the AID C terminus, each of which greatly reduces CSR. Although some of these mutants were previously reported [19,23,24], here we compare the 4 mutants in several assays of AID function. As true for ΔAID, each of the mutations tested reduces binding of UNG to the Sμ region in cells undergoing CSR, although one of the mutants still recruits Msh2. Although ΔAID induces as many Sμ DSBs as FL-AID, all but one of the individual substitutions leads to reduced detection of Sμ DSBs in activated B cells, and the mutants have greatly reduced mutations in the unrearranged Sμ segment relative to cells expressing FL-AID. The phenotypes of the mutants differ from each other, suggesting that these 4 amino acids might interact with different proteins and function in different pathways during CSR.

## Results

### Single amino acid substitutions in the AID C terminus greatly reduce or ablate CSR

**Fig 1:**
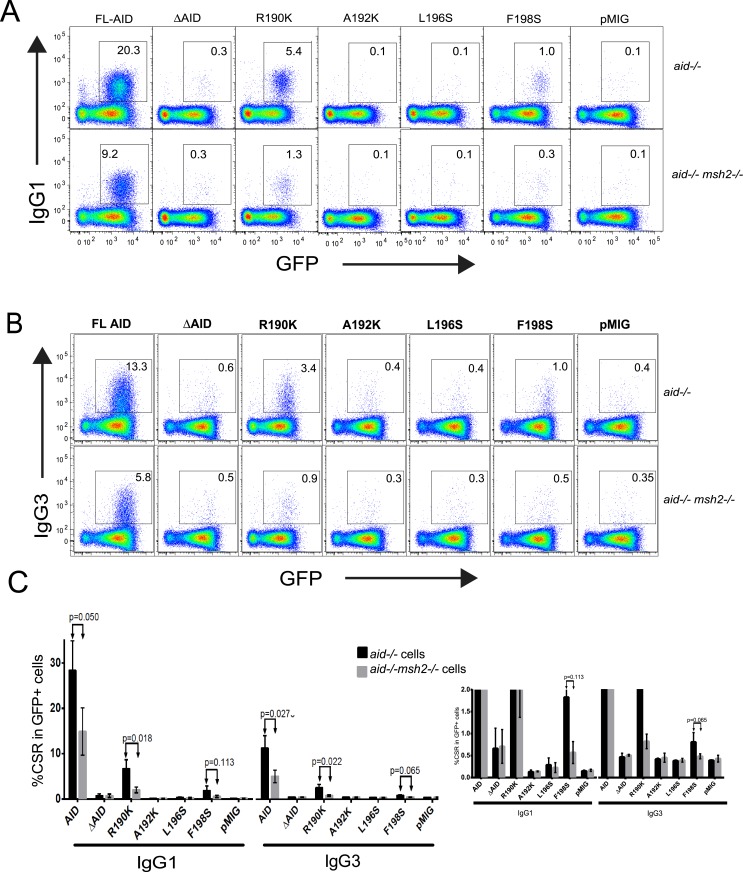
Flow cytometric analysis of CSR to IgG1 and IgG3 by AID C terminus mutants expressed in *aid* ^*-/-*^ cells and in *aid* ^*-/-*^ *msh2* ^*-/-*^ splenic B cells. Live GFP+ cells were gated and CSR was detected with anti-IgG1 (A) or -IgG3 conjugated to PE (B). Shown are results of one representative set of cultures from 3 independent experiments for each retrovirus transduced into *aid* ^*-/-*^ (upper rows) or *aid* ^*-/-*^ *msh2* ^*-/-*^ (lower rows) cells. The numbers in each plot indicate % of GFP+ cells that are IgG1+ (A) or IgG3+ (B). C: Compilation of IgG1 and IgG3 CSR for the C terminus mutants transduced into *aid* ^*-/-*^ and *aid* ^*-/-*^ *msh2* ^*-/-*^ splenic B cells. Duplicate cultures were performed in each experiment, collecting 100,000 events in two experiments and 500,000 in one experiment. Error bars indicate SD. The p values were calculated using an unpaired two-tailed T test. Inset on right shows an expanded scale (up to 2% CSR) of graph on left.

To identify amino acids in the C terminus that are important for CSR, we mutated several amino acids individually and tested their ability to support CSR when expressed in the pMIG retrovirus in *aid* ^*-/-*^ splenic B cells. Cells were activated to switch to IgG1 or IgG3, transduced 24 hrs later with pMIG-AID retroviruses, and harvested two days later. Cell viability was ~70% for all constructs [18]. The viruses express GFP, so to analyze transduced cells we gated on GFP+ cells. Gating strategy is identical to that previously published [18]. Fig 1A and 1B present examples of FACS data demonstrating that specific individual mutations of 4 different amino acids in the AID C terminus greatly reduce CSR to IgG1 and IgG3 relative to that induced by FL/WT-AID. The compiled CSR data for the 4 mutants expressed in *aid* ^*-/-*^ cells are shown in Fig 1C (black bars). Most of the single amino acid mutations reduce CSR by at least 90%, similar to the reduction obtained by deletion of the C terminal 10 amino acids (ΔAID), and similar to previously reported results for ΔAID tagged with the estrogen receptor (ER) [15–17]. The one exception is R190K, which reduces CSR by 75%, although for unknown reasons, the same mutant tagged with ER (R190K-ER) reduces CSR by at least 90% (S1 Fig). A similar reduction in CSR (≥90%) has been reported for two of the mutations in human AID: A192K [23,24] and L198S [19]. In conclusion, 4 different single-amino acid substitution mutations within the C terminal 10 amino acids of AID each individually greatly reduce or abolish CSR.

### Effect of mismatch repair defect on CSR differs among the AID mutants

Previously, we found that Msh2 does not contribute to CSR in cells expressing ΔAID-ER, suggesting that the AID C terminus interacts functionally with Msh2-Msh6 [17]. To determine whether this is also true for CSR in cells expressing the substitution mutants, we transduced these constructs into *aid* ^*-/-*^ *msh2* ^*-/-*^ splenic B cells. As shown in Fig 1C (gray bars), CSR to IgG1 and IgG3 induced by WT AID is reduced by 50–70% in *aid* ^*-/-*^ *msh2* ^*-/-*^ cells relative to *aid* ^*-/-*^ cells, similar to the effect of Msh2 on CSR induced by endogenous AID [33,34], and previous results with transduced AID-ER (S1C Fig) [17]. Also consistent with previous results with ΔAID-ER, IgG1 CSR is not further reduced in *aid* ^*-/-*^ *msh2* ^*-/-*^ cells expressing untagged ΔAID relative to *aid* ^*-/-*^ cells (Fig 1C, see expanded scale insert to the right). IgG3 CSR is at background levels in *aid* ^*-/-*^ cells expressing ΔAID; this is also true for both IgG1 and IgG3 CSR in cells expressing A192K, and L196S. Thus, we cannot determine whether it is reduced in *aid* ^*-/-*^ *msh2* ^*-/-*^ cells. However, CSR induced by the R190K mutant is reduced in cells lacking Msh2, suggesting this mutation does not prevent MMR from contributing to CSR, differing from ΔAID. CSR in cells expressing F198S appears to be reduced in the absence of Msh2, but due to the low CSR level, the difference is not significant.

### Detection of AID C terminus mutants in nuclei and cytoplasm

**Fig 2:**
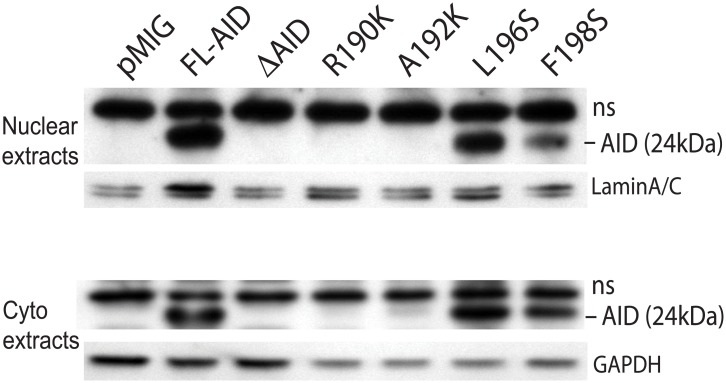
Western blots of nuclear and cytoplasmic extracts from *aid* ^*-/-*^ cells transduced with AID mutants. Anti-AID antibody (directed against the C terminus of AID) [35] was used to detect untagged AID. 80 μg of nuclear and of cytoplasmic extracts expressing AID were analyzed. ns indicates a non-specific band.

As the C terminus is important for AID stability [19,23], it is possible that the reduced CSR was due to low levels of AID. We examined expression of the mutant AID proteins in *aid* ^*-/-*^ cells at the same timepoint used for assaying CSR. In Fig 2, we show expression of the transduced AID mutants detected using rabbit anti-AID antibody directed against the C terminus [35]. The L196S and F198S proteins are well-expressed in both nuclear and cytoplasmic extracts, although F198S is expressed at lower levels than WT AID. We were unable to detect the R190K and A192K mutants in either nuclei or cytoplasmic extracts from *aid* ^*-/-*^ cells using our antibody. However, in a preliminary experiment using a different preparation of antibody also specific for the AID C terminus (from B. Reina-San-Martin), the R190K and A192K mutants were detected almost as well as the L196S mutant, but none of the three mutants were detected as well as WT AID (not depicted). Because the antibodies are directed against the AID C terminus, these experiments are not definitive as to the relative quantity of AID protein in these cells. Nonetheless, the inability of these mutants to induce CSR is probably not explained by their putative instability, because ER- tagged mutant AID proteins are expressed about as well as WT AID and yet induce very little CSR (S1 Fig). We also did not observe any clear difference in the distribution between nuclear and cytoplasmic extracts of the untagged or ER-tagged mutants compared to WT AID. In experiments with ER-tagged AID, cells need to be treated with tamoxifen to induce nuclear localization of AID [17]. Although our nuclear extracts are slightly contaminated with cytoplasmic protein, we do not detect nuclear protein in the cytoplasm (S1B Fig). Taken together, the results suggest that the inability of these mutants to induce CSR is not simply due to their reduced expression or changes in cellular localization.

### Substitution mutants induce fewer Sμ DSBs than AID and ΔAID

**Fig 3:**
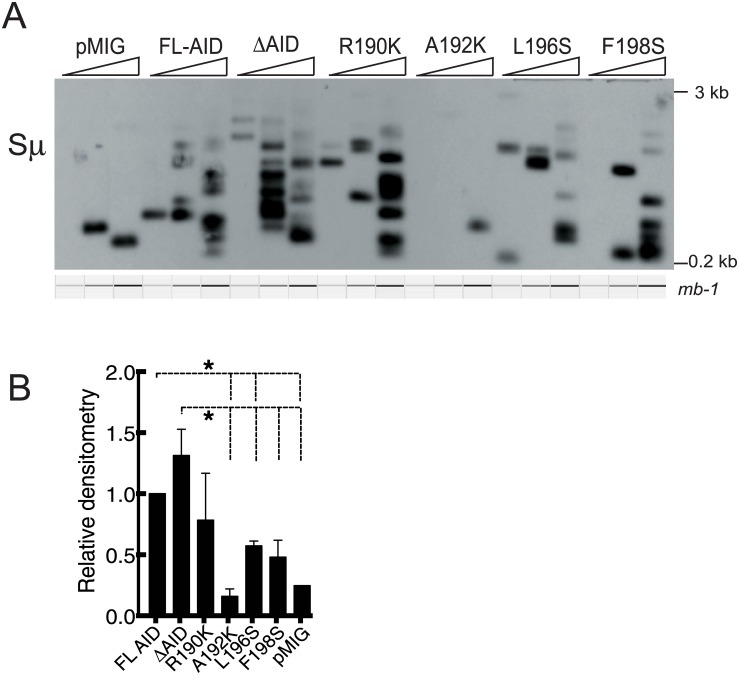
Sμ DSBs are reduced in cells expressing AID C terminus mutants. A: Southern blots of a LM-PCR assay of Sμ DSBs in *aid* ^*-/-*^ cells induced to switch to IgG3 transduced with AID, ΔAID, the indicated mutants, or the empty retrovirus pMIG. RV transduction was performed on day 1 after activation, and cells harvested 2 days later. Three fold titrations of input DNA were assayed, and the *mb-1* gene was amplified as an internal control for template input. The *mb-1* PCR bands shown in A were obtained by electrophoretic analysis on a QIAxcel Advanced instrument, which subjects each sample to electrophoresis in a capillary, and provides an image of each lane. B: Quantitation of the LM-PCR signals by densitometry of all 3 lanes in 3 different experiments, normalized to FL-AID. Error bars = SEM. *p<0.04, determined by two-tailed T test.

To examine the Sμ DSBs induced by AID, ΔAID, and the substitution mutants, we performed ligation-mediated (LM)-PCR on the transduced *aid* ^*-/-*^ cells induced to switch to IgG3. The data shown in Fig 3A are representative of 3 independent experiments. As found previously studying ΔAID-ER [17,18], the frequency of DSBs in Sμ in *aid* ^*-/-*^ cells expressing ΔAID is similar or slightly increased relative to that in cells expressing FL-AID. Surprisingly, cells expressing 3 of the substitution mutants have fewer Sμ DSBs than ΔAID or AID, as shown in the densitometric quantitation of the DSB bands (Fig 3B), where mutants that show significantly reduced DSBs (p<0.04) are indicated by the dotted lines above the histograms. The LM-PCR results suggest that the A192K, L196S, and F198S mutations are each more deleterious to the ability of AID to induce DSBs than the C terminus deletion.

### Recruitment of UNG and Msh2 to Sμ is reduced in cells expressing mutant AID proteins

**Fig 4:**
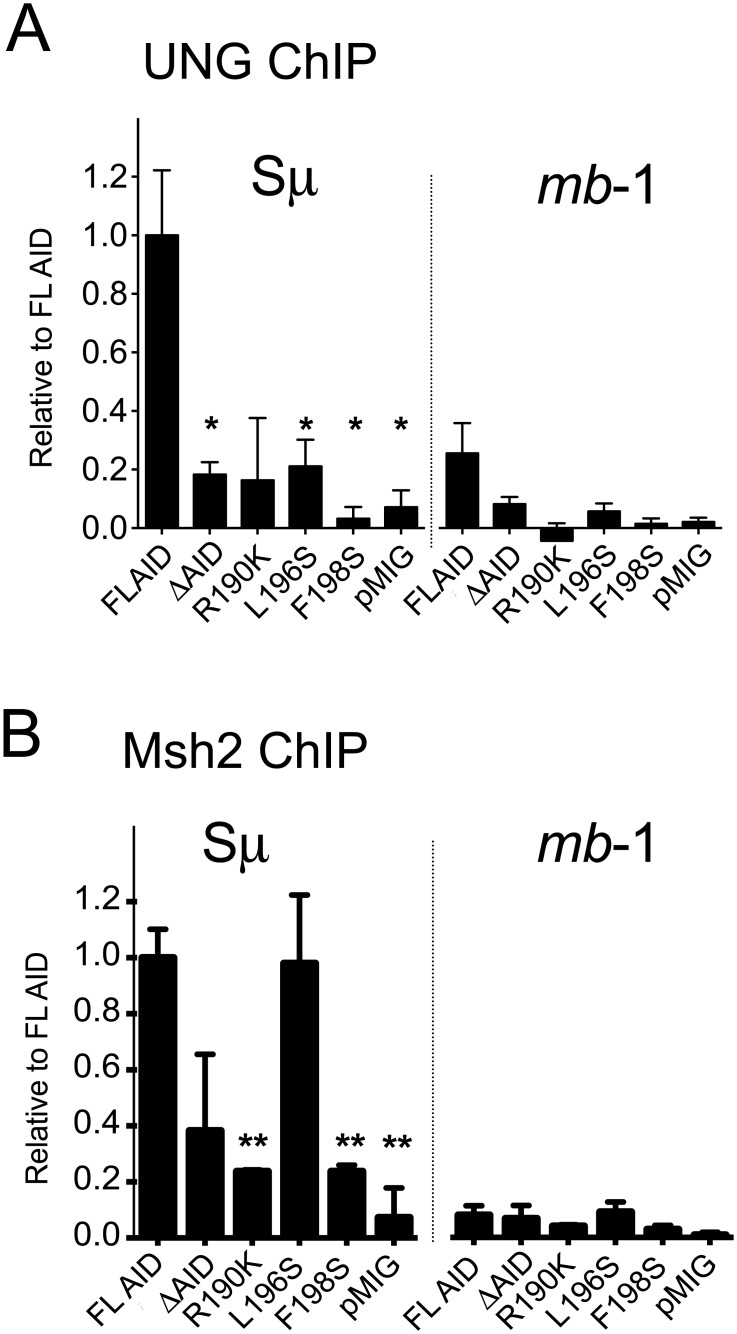
ChIP analyses demonstrate that untagged ΔAID and the substitution mutants poorly recruit UNG and Msh2 (except for L196S) to Sμ. ChIP for UNG (A) and Msh2 (B) at Sμ and at the *mb1* gene in *aid* ^*-/-*^ cells transduced with retroviruses expressing the indicated untagged AID proteins. Cells were induced to switch to IgG3, transduced 1 day after activation, and harvested 2 days later. ChIPs were analyzed by qPCR; % input was calculated, and % input in absence of antibody was subtracted. Results are normalized for each set of mutants to binding results for FL-AID at Sμ for each experiment. Error bars indicate SEM. Three independent experiments with duplicate IPs were performed, except for R190K and F198S, for which one experiment with duplicate IPs was performed, and then the range is indicated. p values for difference from FL-AID were determined by an unpaired 2-tailed T test. *p ≤0.01; **p<0.001. p value for ΔAID in Msh2 ChIP = 0.076.

Using ChIP, we previously showed that AID-ER and ΔAID-ER both bind Sμ [18], and that AID-ER recruits UNG and Msh2 to Sμ and Sγ3 in cells undergoing IgG3 CSR, but ΔAID-ER does not [17]. Also, our ChIP assays showed that AID lacking deaminase activity does not induce UNG or Msh2 to bind to S regions, suggesting that UNG and Msh2-Msh6 are recruited to S regions by interaction with their dU substrates and also with an unknown protein(s) associated with AID, dependent on the AID C terminus [18]. Because ΔAID is a more potent deaminase than WT AID [19,36–38], the reduction in UNG and Msh2-binding observed in cells expressing ΔAID-ER is unlikely to be due to decreased dU bases in Sμ. Here, we examine the ability of untagged FL-AID, ΔAID, and three of the mutants to recruit UNG and Msh2 to Sμ in transduced cells. As shown in Fig 4A none of the mutant AID proteins recruit UNG to Sμ as detected by ChIP. This should result in the introduction of fewer SSBs, consistent with the reduction in DSBs and with the greatly reduced CSR. However, this cannot entirely explain the LM-PCR results, since ΔAID poorly recruits UNG and yet numerous DSBs are detected. Also, as *ung* ^*-/-*^ cells have fewer DSBs than cells expressing these mutant AID proteins [35,39], it is likely that UNG is still able to act on the dU’s introduced by mutant AID, but perhaps less efficiently than in cells expressing WT AID.

Poor recruitment of UNG presumably increases the U:G substrate available for Msh2-Msh6; however, the C terminus mutants also show poor binding of Msh2 to Sμ, except for L196S (Fig 4B). These ChIP results suggest that the substitution mutants might inefficiently introduce SSBs and DSBs into S regions, consistent with the LM-PCR data. However, the finding that Msh2-deficiency decreases CSR in cells expressing R190K as much as it does in cells expressing WT AID suggests that Msh2 is able to act on S regions, despite the fact that Msh2-binding to Sμ was not detected by ChIP. R190K is the mutant that induces the most Sμ DSBs.

### Sequences of S-S junctions demonstrate that AID C terminal mutations impair recombination

Sμ-Sα junctions in cells expressing ΔAID, untagged or tagged, and induced to switch to IgA show evidence of impaired NHEJ, as they have increased lengths of junctional microhomology [18–20,27]. Here we compare Sμ-Sα junctions from *aid* ^*-/-*^ cells expressing untagged AID substitution mutants with junctions from cells expressing FL-AID and ΔAID that were published previously [18], although the cells expressing the substitution mutants were cultured, and the DNA amplified and cloned simultaneously with the cells expressing AID and ΔAID. We chose to study Sμ-Sα junctions rather than Sμ-Sγ junctions because Sα is more homologous to Sμ than are any of the Sγ regions, thus increasing the likelihood of junctional microhomology and the sensitivity of the assay [40]. S2 Fig presents an example of FACS results for IgA CSR. To ensure consistency, we re-analyzed these previously analyzed junctions along with the new sequences. This slightly changed the results, so that although there appears to be an increase in junctional microhomology in the ΔAID samples, it is no longer significant (Table 1).

**Table 1:** Junctional microhomology and inserts at Sμ-Sα junctions in cells expressing AID with C terminal mutations.

Construct (total number of sequences)	Mean junctional microhomology^b^ +/-SEM	Significance^c^ (difference from FL-AID)(p value)	Junctions with inserts (insert lengths)	% Junctions with inserts(difference ^d^ from FL-AID)	% Junctions within Sμ tandem repeats^e^(difference from FL-AID)
FL-AID (56) ^a^	2.16 +/-0.36		6 (1,1,1,2,4,6)	10.7%	5.4%
ΔAID (30) ^a^	4.0 +/-0.84	0.140	2 (1,6)	6.5% (p = 0.475)	40.0% (p = 0.0001)
A192K (1)	15		0	0	0
L196S (31)	6.73 +/-0.94	<0.0001	9 (1,1,1,1,1,1,1,6,6)	29.0% (p = 0.001)	51.6% (p = 0.0001)
F198S (28)	2.26 +/-0.62	0.940	5 (2,2,13,24,25)	17.9% (p = 0.221)	14.9% (p = 0.004)

^a^ These sequences are almost entirely from ref 18, but results are somewhat changed after re-analysis.

^b^ Junctional microhomology data do not include junctions with inserts.

^c^ Significance of difference in lengths of junctional microhomology, using the Mann-Whitney T-test

^d^ Significance of difference in numbers of inserts, using the Chi-square test

^e^ Sμ-Sα junction occurs 3’ (relative to direction of transcription) from position chr12:114,663,504 in the mm9 database on the UCSC browser.

Significance determined using Chi-square test.

Of the substitution mutants for which the S-S junctions were amplified, only the L196S mutation results in a significant and large increase in junctional microhomology. We were unable to amplify junctions from cells expressing the A192K mutant, except for one junction, which had a 15 bp microhomology, consistent with the very few DSBs induced. The F198S mutation did not increase junctional microhomology. The L196S mutant is also the only mutant that showed a significant increase in the frequency of inserts at the Sμ-Sα junction, although the F198S mutant had 3 very large inserts, 13, 24, and 25 bp, much larger than observed with any other AID mutant. Such insertions are another indicator of impaired recombination. Two of the large inserts in junctions from cells expressing F198S were duplicated segments of Sμ and Sα regions, not tandem duplications, nor caused by internal deletions, but instead consistent with fragmentation of the S regions. The third insert matched numerous sites throughout the genome, but not the IgH locus.

### AID C terminal mutations alter the Sμ recombination sites

The position within Sμ where the recombination with the acceptor S region occurs has been shown to differ in several DNA repair mutants from those found in WT cells. For example, in *msh2* ^*-/-*^ and *msh6* ^*-/-*^ mouse B cells, the Sμ junctions occur primarily within the region with a high density of AID hotspots, i.e. the tandem repeat region, whereas in WT cells they occur both within and 5’ to the tandem repeats [34,41]. These data have been interpreted to indicate that without Msh2, the SSBs induced in the 5’ region where there are few AID hotspots are too sparse to form DSBs, as they require Msh2 to be converted into DSBs [3,34,42]. As the C terminus mutants appear to have an impaired ability to induce Sμ DSBs, we asked whether the Sμ-Sα recombination junctions in cells expressing these mutants would be biased towards the Sμ tandem repeat region, which has a very high density of AID hotspots, ~19 per 100 bp. Indeed this is what we found, as shown in Table 1. In WT cells only 5.4% of the junctions occurred in the Sμ tandem repeats, and the remainder were 5’ to the repeats, whereas in cells expressing ΔAID, L196S, or F198S, there was a large increase in the percent of junctions within Sμ tandem repeats. The numbers should not be taken as absolutes, and it is likely that the very low fraction of junctions found in the tandem repeats in cells expressing FL-AID is due to the bias of PCR for amplifying small segments. The upstream primer is located ~355 bp 5’ to the Sμ tandem repeats. Nonetheless, as the PCR’s were performed simultaneously, the results can be compared with each other, and it appears that cells expressing the AID mutants preferentially use Sμ tandem repeats to form Sμ-Sα junctions. This is probably not simply due to the inability of the mutants to recruit Msh2, as the ChIP assay suggests that L196S does recruit Msh2. This, however, could be the explanation for the results for ΔAID and F198S. It is possible that only SSBs within the Sμ tandem repeats are able to generate DSBs that are required for S-S recombination due to the likely low frequency of SSBs induced by these mutants.

### The AID C terminus mutants have reduced germline (GL) Sμ mutations

**Fig 5:**
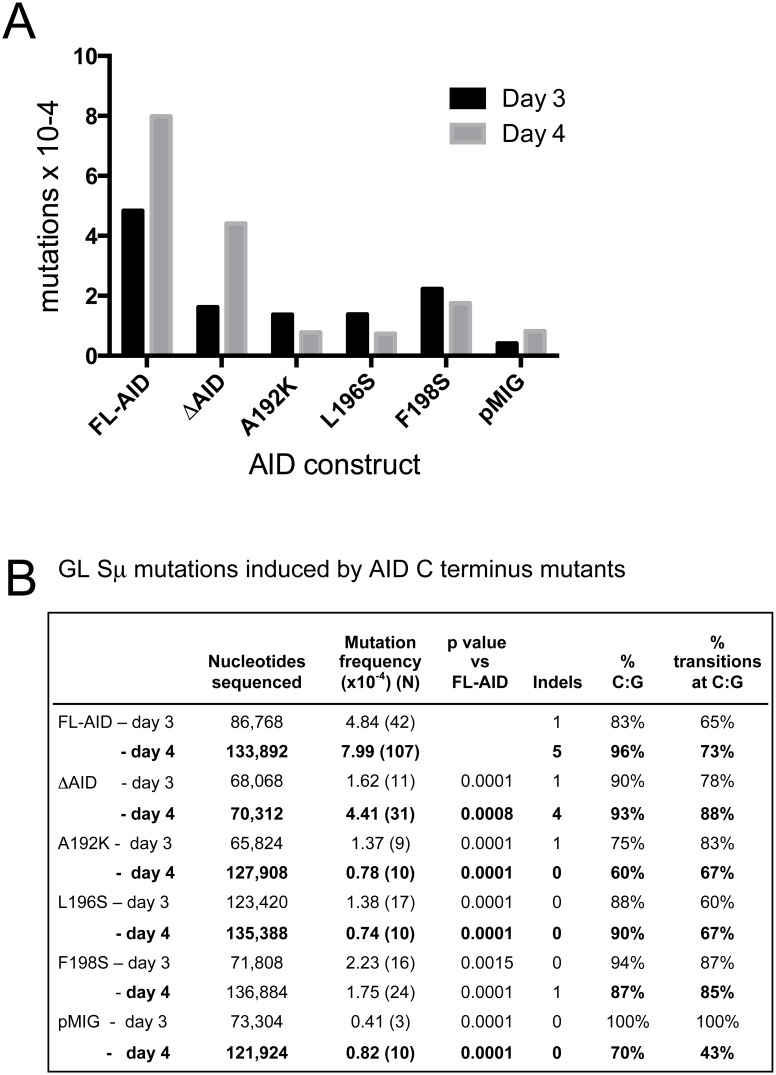
GL 5’Sμ mutations are greatly decreased in *aid* ^*-/-*^ cells induced to switch to IgG3 and expressing AID C terminus mutations. A: Frequency of GL 5’Sμ mutations induced by AID mutants expressed in activated *aid* ^*-/-*^ cells for 3 and 4 days. B: Frequencies and numbers of nucleotide substitutions, p values, indels, % of mutations occurring at C:G bp, % transition mutations at C:G bp, and numbers of nucleotides analyzed within the GL 5’Sμ mutations induced by the AID mutants.

In cells that have been induced to undergo CSR, one can find mutations in the unrearranged/GL 5’Sμ in IgM+ cells. These mutations are due to AID-induced lesions that fail to result in CSR. It is possible that the inability of AID with substitutions in the C terminus to recruit UNG or Msh2 would affect the mutations found in GL 5’Sμ. Previously, it was shown that *aid* ^*-/-*^ cells expressing ΔAID-ER have normal levels of GL 5’Sμ mutations [15]. Here, we sequenced the GL 5’Sμ segment from IgM+ (GFP+) cells purified by FACS to increase the likelihood that the segment analyzed would not be from a switched allele. Although CSR occurs on both the expressed and non-expressed alleles, it tends to be more frequent on the expressed allele [43], and so IgM+ cells are unlikely to have switched on the non-expressed allele. We analyzed mutations on days 3 and 4 after transduction of *aid* ^*-/-*^ cells with untagged FL-AID, ΔAID, the indicated substitution mutants, and pMIG (Fig 5). The cultures had been induced to switch to IgG3. These timepoints are later than we use for assaying CSR and ChIP, or for assaying Sμ DSBs or S-S junctions, which are all analyzed on day 2 after transduction. These later timepoints were used in an attempt to increase the detection of mutations. As expected, there were more Sμ mutations on day 4 than on day 3 in cells expressing WT AID and ΔAID. We find significantly fewer mutations in cells expressing ΔAID relative to cells expressing AID, differing from a previous report [15], perhaps because in the previous study cells were not sorted for being IgM+. On day 3, cells expressing the A192K, L196S, and F198S mutants also have fewer GL 5’Sμ mutations than cells expressing FL-AID, and the frequencies are similar to cells expressing ΔAID. Surprisingly, and unlike cells expressing AID and ΔAID, cells expressing the 3 substitution mutants have fewer mutations on day 4 than on day 3, and the A192K and L196S mutants have as few mutations as cells expressing the pMIG vector. Although we cannot rule it out, the very low GL 5’Sμ mutation frequency detected in cells expressing these mutants is unlikely to be due to decreased deaminase activity, as both ΔAID and the F198S mutant have been reported to have increased deaminase activity [19,32,38,44].

The finding of fewer GL Sμ mutations in cells expressing the AID C terminus substitution mutants is consistent with the reduced frequency of Sμ DSBs found in cells expressing these mutants, but the fact that the mutations decrease with time is surprising. Although the ChIP results indicate that the mutants do not recruit UNG and most do not recruit Msh2 to Sμ, the mutation data do not match GL 5’Sμ mutation results in *ung* ^*-/-*^ *msh2* ^*-/-*^ B cells, which actually have increased S region mutations, rather than decreased mutations [45]. This latter observation is explained by the fact that AID-induced lesions are not repaired and therefore a high frequency of C>T and G>A transitions are observed, along with very few A:T mutations. As shown in Fig 5B, most of the GL 5’Sμ mutations occurred at C:G bp and most were transitions, but this does not appear to increase in cells expressing mutant AID. It is possible that the mutations are reduced at day 4 relative to day 3, because the mutations and DSBs (detected at day 2) are repaired in an error-free manner. Alternatively, it is possible that the AID-induced lesions in these cells are not repaired, and thus destroy the mutated Sμ regions so that they poorly amplify (F198S) or cannot be amplified (L196S) on day 4. For these experiments, viable cells were first isolated by flotation on Lympholyte, and then further purified by FACS based on exclusion of 7-AAD; thus, dead and dying cells are excluded from the analysis. This possibility implies that the only Sμ regions that are present on day 4 in cells transduced with L196S and that can be amplified are those that were not attacked by AID. F198S shows the same trend, although some mutated S regions can be amplified. This interpretation is consistent with the hypothesis that the C terminus of AID is important for repair and recombination processes during CSR.

## Discussion

Deletion of the C terminus prevents AID from being able to induce CSR but has little or no effect on SHM [13–15]. We set out to identify amino acids within the C terminus that are essential for CSR, and to determine if they differentially affect the intermediate steps involved in CSR. The activities previously ascribed to the C terminus include the ability of AID to help recruit UNG and Msh2-Msh6 to S regions, to help recruit proteins involved in NHEJ, e.g. γH2AX, Nbs1, ATM, 53BP1, Ku, XRCC4, and DNA-PKcs to S regions, and to inhibit end-resection which would prevent NHEJ [17–20,26,27]. Although previously there was no evidence that the C terminus affects mutations or DSBs in Sμ, we report here that the C terminus also affects these two intermediate steps in CSR.

We characterized 4 substitution mutations within the C terminus of mouse AID that each greatly reduce or ablate the ability of AID to induce CSR when transduced into *aid* ^*-/-*^ splenic B cells. Except for their effect on CSR, none of the 4 mutants have the same phenotype as a deletion of the 10 amino acids of the AID C terminus, and all, except R190K, appear to have more deleterious effects on some of the intermediate steps involved in CSR than does a C terminus deletion. The more severe effects might be because these particular mutations are at sites important for interaction with other proteins, and because they also involve substitution of amino acids with very different properties from those in the WT protein, thus possibly interfering with interactions between AID and other proteins more than does a deletion. As each mutant has a distinct phenotype, this suggest that the AID C terminus might interact with several proteins, each of which is important for AID function. Table 2 summarizes the properties of the individual mutants.

**Table 2:** Characteristics of mutant AID proteins.

	CSR	Sμ DSBs	ChIP UNG	ChIP Msh2	Mutations in GL SμDay 3	Mutations in GL SμDay 4	Sμ-Sα junctions(microhomology)
FL-AID	+++	+++	+++	+++	++	+++	
ΔAID	-	+++	-	+	+	++	marginal^b^ **↑**
R190K	+	+++	-	-	ND^a^	ND	ND
A192K	-	-	ND ^a^	ND	+	-	**↑** ^c^
L196S	-	++	-	+++	+	-	**↑**
F198S	-	++	-	-	+	+	not changed

^a^ Not done

^b^ Not significant

^c^ Only 1 junction could be amplified

Two of the mutations, L196S and F198S, should prevent interaction with Crm1, a protein that specifically interacts with the C terminus of AID and promotes nuclear export of AID [21,23,24]. Crm1 binding was reported to depend upon hydrophobic amino acids at positions 189, 193, 196 and 198 in human AID [24]. Mutation of F198 to Ala in mouse AID was shown to eliminate Crm1 binding, cause an increase in the proportion of AID in nuclei, and to reduce CSR in splenic B cells by 40% [21], whereas we find that a different mutation at this amino acid (F198S) reduces CSR by >90% and doesn’t seem to affect localization of untagged AID. This is consistent with reports that there is no correlation between Crm1-binding or nuclear export and the ability of AID to support CSR [23,24]. Nuclear export stabilizes AID, as AID is ~3-times as stable in the cytoplasm as in the nucleus [22,46]. However, we find that L196S and F198S are expressed only slightly less well than unmutated AID, and show normal ratios of proteins in the nucleus versus cytoplasm (Fig 2). Thus, the dramatic reductions in CSR caused by the L196S and F198S mutations cannot be due to effects on nuclear-cytoplasmic distribution, or effects on AID stability. Although neither of these two mutants causes detectable binding of UNG to Sμ in our ChIP assays, the mutants differ in their ability to stabilize binding of Msh2 to Sμ, and differ in their effect on S-S junctions. L196S increases the lengths of junctional microhomology, and increases the frequency of inserts at junctions, whereas the F198S mutation does not.

The other two mutations (R190K and A192K) are at sites not known to interact with other proteins. R190K induces ~30% CSR relative to WT AID, and about as many Sμ DSBs as in cells expressing WT AID. Human AID-A192K was previously shown to be unstable, although it was shown to bind Crm1 [24]. Although it is possible these proteins are less stable than WT AID, attachment of an ER tag to these two mutants caused them to be well-expressed in both nuclei and cytoplasm in the presence of tamoxifen, although they still could not induce CSR (S1 Fig).

It is entirely possible that the increased junctional microhomology or increased junctional inserts observed are not only due to problems recruiting NHEJ proteins *per se*, but also due to problems producing DSBs appropriate for NHEJ, which might in turn prevent recruitment of NHEJ proteins [47]. Poor recruitment of UNG and MMR likely decreases the rate of introduction of SSBs and DSBs, reducing the generation of blunt DSBS that are the substrate for NHEJ [4]. Thus, DSBs might have relatively long ss tails, which are poor substrates for NHEJ. DSBs might therefore be limiting, delaying recombination and allowing end-resection to occur, resulting in the use of microhomologies to stabilize the junctions. Evidence consistent with these possibilities is the finding that *ung* ^*-/-*^, *mlh1* ^*-/-*^, and *pms2* ^*-/-*^ cells, each of which have few Sμ DSBs [6,35,39], all show increased S-S junctional microhomology [6,27,40].

The most surprising and interesting result is our finding that GL 5’Sμ mutations are greatly reduced in cells expressing ΔAID or the substitution mutants, relative to WT AID. Three days after transduction, GL Sμ mutations were lower in cells expressing ΔAID or the substitution mutants than in cells expressing WT AID. This does not appear to be explained by reduced catalytic activity, as both ΔAID and F198S have been shown to have the same or increased deaminase activity relative to WT AID [19,32,37,38], although the other 3 mutants have not been tested. Most striking was the finding that mutations decreased between days 3 and 4 in cells expressing the A192K, L196S and F198S mutants, and were actually reduced to background level in cells expressing the A192K and L196S mutants, i.e. only unmutated GL 5’Sμ segments could be recovered on day 4. These results could be due to error-free repair of the mutations and DSBs induced in cells expressing these AID mutants, although several reports indicate that ΔAID is unable to recruit or support the binding of DNA repair proteins to the Sμ region. Thus, it appears more likely that cells expressing these mutants are unable to repair or recombine the AID-induced lesions, resulting in destruction of these Sμ segments, so that they cannot be amplified by PCR, and so that only Sμ regions that had not been targeted by AID could be amplified. This hypothesis is consistent with a previous report that cells expressing ΔAID accumulate DNA damage [19].

We hypothesize that the C terminus is not only important for S-S recombination itself, but also for repairing unrecombined DSBs within Sμ, consistent with the reported poor recruitment of NHEJ proteins. However, poor recruitment of NHEJ proteins does not by itself explain the lack of CSR, as cells without NHEJ proteins can still undergo substantial CSR [28]. ΔAID (human and mouse) and the human AID mutant L198S are dominant-negative mutants [14,18,19,48]. The possibility that the C terminus substitution mutants result in destruction of AID-targeted Sμ regions could explain the dominant-negative activity, because if mutant AID destroys S regions in cells expressing WT AID, then WT AID would be rendered non-functional, as there would be no S regions to act upon. This hypothesis is further supported by the fact that deaminase activity is required for the dominant-negative activity ofΔAID [18,19].

## Materials and Methods

### Mice

This study was approved by, and performed in according with the guidelines provided by, the University of Massachusetts Medical School Animal Care and Use Committee. Mice were housed in a pathogen-free facility. AID and Msh2-deficient mice were previously described [49,50], and were generated by breeding heterozygotes. Mice were sacrificed by CO_2_ asphyxiation followed by bilateral pneumothorax.

### Antibodies

Antibodies to ER (sc-8002X), GAPDH (sc-25778), and Msh2 (sc-494) were purchased from Santa Cruz, and antibody for Lamin A/C was from Cell Signaling (#2032). Rabbit antibodies to mouse AID [35] and UNG [6] were previously described.

### Retroviruses

pMX-PIE-AID-FLAG-ER-IRES-GFP-*puro* and pMX-PIE-ΔAID-FLAG-ER-IRES-GFP-*puro* [15] were received from Drs V. Barretto and M. Nussenzweig (The Rockefeller University, NY). The control retrovirus pMX-PIE-ER-IRES-GFP was constructed and viruses were prepared as previously described [17]. AID-pMIG and pMIG [51] were received from Dr J. Chaudhuri (Sloan-Kettering Memorial Cancer Center, NY). To create the AID substitution mutants, the AID gene was subcloned into Bluescript (Stratagene), mutated using Quik-Change (Stratagene), sequenced, and then reinserted into pMX-PIE and pMIG. pMIG-ΔAID was created by converting amino acid position 189 to a nonsense codon. Retroviruses were produced in Phoenix-E cells.

### B cell purification, cultures and CSR

Briefly, purified splenic B cells were activated to switch to IgG3, IgG1, or IgA for 1 day, and then infected with pMIG retroviruses [18]. To induce IgA CSR, cells were activated with TGF-β (2 ng/ml), IL-5 (1.5 ng/ml), anti-δ-dextran (10 ng/ml) (Fina BioSolutions, Rockville MD), and retinoic acid (10 nM). For most assays, including analysis of CSR, cells infected with pMIG retroviruses were harvested two days after infection. For the mutation analysis, cells were harvested 3 and 4 days after infection. Cells were maintained in these longer cultures by dilution and additional feeding. Cells infected with retroviruses expressing ER-tagged AID were treated with tamoxifen at the time of infection (2 days after activation), and harvested one day later [17]. To assay CSR, cells were stained with 7-AAD and F(ab’)_2_ Ab to IgG1, IgG3, or intact antibody specific for IgA (conjugated to phycoerythrin (PE)) (Southern Biotech), and analyzed by flow cytometry on a MACSQuant analyzer (Miltenyi Biotec). Compensation and gating were performed using FlowJo software (TreeStar), as shown in [18]. Dead/dying (7AAD+) cells were excluded, and live, single GFP+ cells were gated for analysis of CSR.

### Western blotting

Preparation of extracts and western blots were previously described [5].

### Ligation-mediated PCR (LM-PCR), chromatin immunoprecipitation (ChIP) and amplification and sequencing of Sμ-Sα junctions

They were performed as previously described [18].

### Amplification and sequencing of 5’ GL Sμ

*Aid* ^*-/-*^ splenic B cells were activated to switch to IgG3 and infected after one day with unmutated or mutated AID-pMIG or empty vector, pMIG. Four independent cultures for each retroviral (RV) infection were set up, and 2 days after infection, cultures were split and fed with fresh medium and switch inducers. Viable cells were isolated by flotation on Lympholyte 3 and 4 days after infection, and GFP+IgM+ cells from each culture were sorted independently on a FACSAria (Becton Dickson) after staining with anti-IgM-PE (Southern Biotech) and 7AAD. Two experiments were performed for each RV-AID analyzed, except for ΔAID analyzed on days 3 and 4, and A192K and F198S analyzed on day 3, where one experiment was performed for each. PfuTurbo (Agilent) (error rate = 1.3 x 10^−6^) was used to amplify a 748 bp 5’ GL Sμ fragment. Primers were: 5u3 (forward primer) 5’-AATGGATACCTCAGTGGTTTTTAATGGTGGGTTTA-3’ [52] and m2R (reverse primer) 5’-GCTACTCCAGAGTATCTCATTTCAGATC-3’ [45]. Significance of difference of mutation frequency from FL-AID was determined by a chi-square test.

## Supporting Information

S1 Fig Western blots and flow cytometric analysis of CSR to IgG1 and IgG3 by AID-ER C terminus mutants expressed in *aid* ^*-/-*^ cells and *aid* ^*-/-*^ *msh2* ^*-/-*^ splenic B cells. **A:** Western blots, probed with anti-ER antibody, indicate that the AID-ER mutants were expressed about as well as FL/WT AID-ER in *aid* ^*-/-*^ cells. Since the C terminus has a nuclear export signal, by adding the ER tag, we can induce nuclear localization of AID by adding tamoxifen to the cells, thus neutralizing the effect of loss of the nuclear export signal. AID tagged with ER forms 3 bands (~55, 62, and 85 kDa) in nuclei but two bands in the cytoplasm, although ΔAID also shows a smaller fragment. The 85 kDa band might be due to dimerization through the ER tag, as the ER tag expressed alone also forms two bands. In this blot, we examined a double mutant, R190K-A192K-ER, but not the R190K-ER mutant. In a separate experiment, we found that R190K-ER is expressed as well as AID-ER (not depicted). 7 μg of nuclear extracts and 20 μg of cytoplasmic extracts expressed tagged AID were analyzed. These proteins cannot be detected with our polyclonal AID antibody, as the ER tag blocks the C terminus epitopes. **B**: Western blots to assay purity of the nuclear and cytoplasmic splenic B cell extracts. Western blots of nuclear extracts (lanes 1–3)(40 μg/lane) and cytoplasmic extracts (lanes 4–6) (75 μg/lane) from RV-transduced B cells incubated with antibodies to Lamin A/C, specific for nuclei, and with GAPDH, specific for cytoplasm. Lanes 1, 4—extracts from cells transduced with AID-ER; lanes 2, 5 –extracts from cells transduced with ΔAID-ER; lanes 3,6 –extracts from cells transduced with ER. Nuclear and cytoplasmic blots were incubated together with each antibody, washed, and exposed simultaneously to the same film. The mol wt markers indicated are the ones closest in size to the indicated proteins. **C**: CSR induced by AID-ER constructs. Cells were induced to switch to IgG1 or IgG3, as indicated, and transduced with the indicated AID constructs on day 2, and cells were harvested and assayed on day 3. Three independent experiments with duplicate cultures were performed, collecting 100,000 events in two experiments and 500,000 in one experiment. Error bars indicate SD. The p values were calculated using an unpaired two-tailed T test. Although not shown, AID-F198S-ER switched to IgG1 and IgG3 as well as WT-AID-ER.
(EPS)

S2 Fig Example of FACS results for CSR to IgA in cells transduced with untagged AID mutants in pMIG. Live GFP+ cells were gated and CSR was detected with anti-IgA conjugated to PE 2 days after infection. Shown are results of one representative set of cultures from 2 independent experiments for each retrovirus transduced into *aid* ^*-/-*^ cells. The numbers in each plot indicate % of GFP+ cells that are IgA+.
(EPS)

## References

[1] Petersen-Mahrt SK, Harris RS, Neuberger MS (2002) AID mutates E. coli suggesting a DNA deamination mechanism for antibody diversification. Nature 418: 99–104. 12097915 10.1038/nature00862

[2] Rada C, Williams GT, Nilsen H, Barnes DE, Lindahl T, Neuberger MS (2002) Immunoglobulin isotype switching is inhibited and somatic hypermutation perturbed in UNG-deficient mice. Curr Biol 12: 1748–1755. 12401169 10.1016/s0960-9822(02)01215-0

[3] Stavnezer J, Guikema JEJ, Schrader CE (2008) Mechanism and regulation of class switch recombination. Ann Rev Immunol 26: 261–292. 18370922 10.1146/annurev.immunol.26.021607.090248 PMC2707252

[4] Stavnezer J, Schrader CE (2014) IgH Chain Class Switch Recombination: Mechanism and Regulation. J Immunol 193: 5370–5378. 10.4049/jimmunol.1401849 25411432 PMC4447316

[5] Guikema JE, Linehan EK, Tsuchimoto D, Nakabeppu Y, Strauss PR, Stavnezer J, et al (2007) APE1- and APE2-dependent DNA breaks in immunoglobulin class switch recombination. J Exp Med 204: 3017–3026. 18025127 10.1084/jem.20071289 PMC2118529

[6] Schrader CE, Guikema JE, Linehan EK, Selsing E, Stavnezer J (2007) Activation-induced cytidine deaminase-dependent DNA breaks in class switch recombination occur during G1 phase of the cell cycle and depend upon mismatch repair. J Immunol 179: 6064–6071. 17947680 10.4049/jimmunol.179.9.6064

[7] Pena-Diaz J, Bregenhorn S, Ghodgaonkar M, Follonier C, Artola-Boran M, Castor D, et al (2012) Noncanonical mismatch repair as a source of genomic instability in human cells. Mol Cell 47: 669–680. 10.1016/j.molcel.2012.07.006 22864113

[8] Stavnezer J, Linehan EK, Thompson MR, Habboub G, Ucher AJ, Kadungure T, et al (2014) Differential expression of APE1 and APE2 in germinal centers promotes error-prone repair and A:T mutations during somatic hypermutation. Proc Natl Acad Sci U S A 111: 9217–9222. 10.1073/pnas.1405590111 24927551 PMC4078814

[9] Rada C, Di Noia JM, Neuberger MS (2004) Mismatch recognition and uracil excision provide complementary paths to both Ig switching and the A/T-focused phase of somatic mutation. Mol Cell 16: 163–171. 15494304 10.1016/j.molcel.2004.10.011

[10] Peled JU, Kuang FL, Iglesias-Ussel MD, Roa S, Kalis SL, Goodman MF, et al (2008) The biochemistry of somatic hypermutation. Annu Rev Immunol 26: 481–511. 10.1146/annurev.immunol.26.021607.090236 18304001

[11] Maul RW, Gearhart PJ (2010) AID and somatic hypermutation. Adv Immunol 105: 159–191. 10.1016/S0065-2776(10)05006-6 20510733 PMC2954419

[12] Dingler FA, Kemmerich K, Neuberger MS, Rada C (2014) Uracil excision by endogenous SMUG1 glycosylase promotes efficient Ig class switching and impacts on A:T substitutions during somatic mutation. Eur J Immunol. 10.1002/eji.201444482 PMC4158878 24771041

[13] Revy P, Muto T, Levy Y, Geissmann F, Plebani A, Sanal O, et al (2000) Activation-induced cytidine deaminase (AID) deficiency causes the autosomal recessive form of the Hyper-IgM syndrome (HIGM2). Cell 102: 565–575. 11007475 10.1016/s0092-8674(00)00079-9

[14] Ta VT, Nagaoka H, Catalan N, Durandy A, Fischer A, Imai K, et al (2003) AID mutant analyses indicate requirement for class-switch-specific cofactors. Nat Immunol 4: 843–848. 12910268 10.1038/ni964

[15] Barreto V, Reina-San-Martin B, Ramiro AR, McBride KM, Nussenzweig MC (2003) C-terminal deletion of AID uncouples class switch recombination from somatic hypermutation and gene conversion. Mol Cell 12: 501–508. 14536088 10.1016/s1097-2765(03)00309-5

[16] Doi T, Kato L, Ito S, Shinkura R, Wei M, Nagaoka H (2009) The C-terminal region of activation-induced cytidine deaminase is responsible for a recombination function other than DNA cleavage in class switch recombination. Proc Natl Acad Sci U S A 106: 2758–2763. 10.1073/pnas.0813253106 19202055 PMC2650339

[17] Ranjit S, Khair L, Linehan EK, Ucher AJ, Chakrabarti M, Schrader CE, et al (2011) AID binds cooperatively with UNG and Msh2-Msh6 to Ig switch regions dependent upon the AID C terminus. J Immunol 187: 2464–2475. 10.4049/jimmunol.1101406 21804017 PMC3159830

[18] Ucher AJ, Ranjit S, Kadungure T, Linehan EK, Khair L, Xie E, et al (2014) Mismatch Repair Proteins and AID Activity Are Required for the Dominant Negative Function of C-Terminally Deleted AID in Class Switching. J Immunol 193: 1440–1450. 10.4049/jimmunol.1400365 24973444 PMC4142545

[19] Zahn A, Eranki AK, Patenaude AM, Methot SP, Fifield H, Lamarre A, et al (2014) Activation induced deaminase C-terminal domain links DNA breaks to end protection and repair during class switch recombination. Proc Natl Acad Sci U S A 111: E988–997. 10.1073/pnas.1320486111 24591601 PMC3964074

[20] Sabouri S, Kobayashi M, Begum NA, Xu J, Hirota K, Honjo T (2014) C-terminal region of activation-induced cytidine deaminase (AID) is required for efficient class switch recombination and gene conversion. Proc Natl Acad Sci U S A 111: 2253–2258. 10.1073/pnas.1324057111 24469810 PMC3926049

[21] McBride KM, Barreto V, Ramiro AR, Stavropoulos P, Nussenzweig MC (2004) Somatic hypermutation is limited by CRM1-dependent nuclear export of activation-induced deaminase. J Exp Med 199: 1235–1244. 15117971 10.1084/jem.20040373 PMC2211910

[22] Aoufouchi S, Faili A, Zober C, D'Orlando O, Weller S, Weill JC, et al (2008) Proteasomal degradation restricts the nuclear lifespan of AID. J Exp Med 205: 1357–1368. 10.1084/jem.20070950 18474627 PMC2413033

[23] Geisberger R, Rada C, Neuberger MS (2009) The stability of AID and its function in class-switching are critically sensitive to the identity of its nuclear-export sequence. Proc Natl Acad Sci U S A 106: 6736–6741. 10.1073/pnas.0810808106 19351893 PMC2672500

[24] Ellyard JI, Benk AS, Taylor B, Rada C, Neuberger MS (2011) The dependence of Ig class-switching on the nuclear export sequence of AID likely reflects interaction with factors additional to Crm1 exportin. Eur J Immunol 41: 485–490. 10.1002/eji.201041011 21268017 PMC3437479

[25] Ranjit S, Khair L, Linehan EK, Ucher AJ, Chakrabarti M, Schrader CR, et al (2014) Correction: AID Binds Cooperatively With UNG and Msh2-Msh6 to Ig Switch Regions Dependent upon the AID C Terminus. J Immunol 192: 4934. 10.4049/jimmunol.1101406 PMC3159830 21804017

[26] Wu X, Geraldes P, Platt JL, Cascalho M (2005) The double-edged sword of activation-induced cytidine deaminase. J Immunol 174: 934–941. 15634916 10.4049/jimmunol.174.2.934

[27] Kracker S, Imai K, Gardes P, Ochs HD, Fischer A, Durandy A (2010) Impaired induction of DNA lesions during immunoglobulin class-switch recombination in humans influences end-joining repair. Proc Natl Acad Sci U S A 107: 22225–22230. 10.1073/pnas.1012591108 21135220 PMC3009794

[28] Boboila C, Yan C, Wesemann DR, Jankovic M, Wang JH, Manis J, et al (2010) Alternative end-joining catalyzes class switch recombination in the absence of both Ku70 and DNA ligase 4. J Exp Med 207: 417–427. 10.1084/jem.20092449 20142431 PMC2822597

[29] Cortizas EM, Zahn A, Hajjar ME, Patenaude AM, Di Noia JM, Verdun RE (2013) Alternative End-Joining and Classical Nonhomologous End-Joining Pathways Repair Different Types of Double-Strand Breaks during Class-Switch Recombination. J Immunol 191: 5751–5763. 10.4049/jimmunol.1301300 24146042

[30] Jeevan-Raj BP, Robert I, Heyer V, Page A, Wang JH, Cammas F, et al (2011) Epigenetic tethering of AID to the donor switch region during immunoglobulin class switch recombination. J Exp Med 208: 1649–1660. 10.1084/jem.20110118 21746811 PMC3149220

[31] Vuong BQ, Herrick-Reynolds K, Vaidyanathan B, Pucella JN, Ucher AJ, Donghia NM, et al (2013) A DNA break- and phosphorylation-dependent positive feedback loop promotes immunoglobulin class-switch recombination. Nat Immunol 14: 1183–1189. 10.1038/ni.2732 24097111 PMC4005274

[32] Patenaude AM, Orthwein A, Hu Y, Campo VA, Kavli B, Buschiazzo A, et al (2009) Active nuclear import and cytoplasmic retention of activation-induced deaminase. Nat Struct Mol Biol 16: 517–527. 10.1038/nsmb.1598 19412186

[33] Schrader CE, Edelmann W, Kucherlapati R, Stavnezer J (1999) Reduced isotype switching in splenic B cells from mice deficient in mismatch repair enzymes. J Exp Med 190: 323–330. 10430621 10.1084/jem.190.3.323 PMC2195591

[34] Ehrenstein MR, Neuberger MS (1999) Deficiency in Msh2 affects the efficiency and local sequence specificity of immunoglobulin class-switch recombination: parallels with somatic hypermutation. Embo J 18: 3484–3490. 10369687 10.1093/emboj/18.12.3484 PMC1171427

[35] Schrader CE, Linehan EK, Mochegova SN, Woodland RT, Stavnezer J (2005) Inducible DNA breaks in Ig S regions are dependent upon AID and UNG. J Exp Med 202: 561–568. 16103411 10.1084/jem.20050872 PMC2212854

[36] Bransteitter R, Pham P, Calabrese P, Goodman MF (2004) Biochemical analysis of hypermutational targeting by wild type and mutant activation-induced cytidine deaminase. J Biol Chem 279: 51612–51621. 15371439 10.1074/jbc.M408135200

[37] Kohli RM, Abrams SR, Gajula KS, Maul RW, Gearhart PJ, Stivers JT, et al (2009) A Portable Hot Spot Recognition Loop Transfers Sequence Preferences from APOBEC Family Members to Activation-induced Cytidine Deaminase. J Biol Chem 284: 22898–22904. 10.1074/jbc.M109.025536 19561087 PMC2755697

[38] Mu Y, Prochnow C, Pham P, Chen XS, Goodman MF (2012) A structural basis for the biochemical behavior of activation-induced deoxycytidine deaminase class-switch recombination-defective hyper-IgM-2 mutants. J Biol Chem 287: 28007–28016. 10.1074/jbc.M112.370189 22715099 PMC3431652

[39] Imai K, Slupphaug G, Lee WI, Revy P, Nonoyama S, Catalan N, et al (2003) Human uracil-DNA glycosylase deficiency associated with profoundly impaired immunoglobulin class-switch recombination. Nat Immunol 4: 1023–1028. 12958596 10.1038/ni974

[40] Stavnezer J, Bjorkman A, Du L, Cagigi A, Pan-Hammarstrom Q (2010) Mapping of switch recombination junctions, a tool for studying DNA repair pathways during immunoglobulin class switching. Adv Immunol 108: 45–109. 10.1016/B978-0-12-380995-7.00003-3 21056729

[41] Li Z, Scherer SJ, Ronai D, Iglesias-Ussel MD, Peled JU, Bardwell PD, et al (2004) Examination of Msh6- and Msh3-deficient mice in class switching reveals overlapping and distinct roles of MutS homologues in antibody diversification. J Exp Med 200: 47–59. 15238604 10.1084/jem.20040355 PMC2213317

[42] Min I, Schrader C, Vardo J, D'Avirro N, Luby T, Stavnezer J, et al (2003) The Sμ tandem repeat region is critical for isotype switching in the absence of Msh2. Immunity 19: 515–524. 14563316 10.1016/s1074-7613(03)00262-0

[43] Hummel M, Kaminshka J, Dunnick W (1987) Switch region content of hybridomas: the two spleen cell IgH loci tend to rearrange to the same isotype. J Immunol 138: 3539–3548. 3106486

[44] Larijani M, Martin A (2012) The biochemistry of activation-induced deaminase and its physiological functions. Semin Immunol 12: 255–263. 10.1016/j.smim.2012.05.003 22695318

[45] Xue K, Rada C, Neuberger MS (2006) The in vivo pattern of AID targeting to immunoglobulin switch regions deduced from mutation spectra in msh2-/- ung-/- mice. J Exp Med 203: 2085–2094. 16894013 10.1084/jem.20061067 PMC2118391

[46] Orthwein A, Patenaude AM, Affar el B, Lamarre A, Young JC, Di Noia J (2010) Regulation of activation-induced deaminase stability and antibody gene diversification by Hsp90. J Exp Med 207: 2751–2765. 10.1084/jem.20101321 21041454 PMC2989769

[47] Pannunzio NR, Li S, Watanabe G, Lieber MR (2014) Non-homologous end joining often uses microhomology: implications for alternative end joining. DNA Repair (Amst) 17: 74–80. 24613510 10.1016/j.dnarep.2014.02.006 PMC4440676

[48] Imai K, Zhu Y, Revy P, Morio T, Mizutani S, Fishcer A, et al (2005) Analysis of class switch recombination and somatic hypermutation in patients affected with autosomal dominant hyper-IgM syndrome type 2. Clin Immunol 115: 277–285. 15893695 10.1016/j.clim.2005.02.003

[49] Muramatsu M, Kinoshita K, Fagarasan S, Yamada S, Shinkai Y, Honjo T (2000) Class switch recombination and hypermutation require activation-induced cytidine deaminase (AID), a potential RNA editing enzyme. Cell 102: 553–563. 11007474 10.1016/s0092-8674(00)00078-7

[50] Reitmair AH, Schmits R, Ewel A, Bapat B, Redston M, Mitri A, et al (1995) MSH2 deficient mice are viable and susceptible to lymphoid tumours. Nat Genet 11: 64–70. 7550317 10.1038/ng0995-64

[51] Vuong BQ, Lee M, Kabir S, Irimia C, Macchiarulo S, McKnight GS, et al (2009) Specific recruitment of protein kinase A to the immunoglobulin locus regulates class-switch recombination. Nat Immunol 10: 420–426. 10.1038/ni.1708 19234474 PMC4169875

[52] Schrader CE, Bradley SP, Vardo J, Mochegova SN, Flanagan E, Stavnezer J (2003) Mutations occur in the Ig Sμ region but rarely in Sγ regions prior to class switch recombination. Embo J 22: 5893–5903. 14592986 10.1093/emboj/cdg550 PMC275407

